# The protective effects of S14G-humanin (HNG) against streptozotocin (STZ)-induced cardiac dysfunction

**DOI:** 10.1080/21655979.2021.1964894

**Published:** 2021-09-10

**Authors:** Xiaopan Chen, Chuan Yun, Hailong Zheng, Xu Chen, Qianfei Han, Hua Pan, Yang Wang, Jianghua Zhong

**Affiliations:** ^a^. Department Of Endocrinology, The First Affiliated Hospital of Hainan Medical University, Hainan, China; ^b^. Department Of Cardiology, Affiliated Haikou Hospital Of Xiangya Medical College, Central South University, Haikou, Hainan Province, China

**Keywords:** S14G-humanin, streptozotocin, cardiac dysfunction, diabetes

## Abstract

Excessive oxidative stress, inflammation, and myocardial hypertrophy have been associated with diabetic cardiomyopathy (DCM). S14G-humanin (HNG) is a potent humanin analogue that has demonstrated cytoprotective effects in a variety of cells and tissues. However, the pharmacological function of HNG in diabetic cardiomyopathy has not yet been reported. In the present study, we investigated the protective effects of HNG against streptozotocin (STZ)-induced cardiac dysfunction in diabetic mice. Myocardial hypertrophy in diabetic mice was determined using Wheat Gem Agglutinin (WGA) staining. The heart function was measured with Echocardiographic imaging. Levels of tumor necrosis factor-α (TNF-α) and interleukin-6 (IL-6) proteins in plasma were measured using enzyme-linked immunosorbent assay (ELISA) kits. Protein expression of Phosphorylated p38/p38 was determined using western blots. We found that HNG treatment attenuated the STZ-induced myocardial hypertrophy and significantly improved heart function. Also, its treatment proved effective as it reduced the levels of several myocardial injury indicators, including creatine kinase-MB (CK-MB), aspartate aminotransferase (AST), lactate dehydrogenase (LDH), and both the cardiac and plasma levels of TNF-α and IL-6, highlighting its effect on the STZ-induced myocardial injury. Lastly, HNG suppressed the activation of the p38/nuclear factor kappa-B (NF-κB) signaling pathway. S14G humanin possesses protective effects against streptozotocin-induced cardiac dysfunction through inhibiting the activation of the p38/NF-κB signaling pathway.

## 1. Introduction

The prevalence of type 2 diabetes mellitus (T2DM) has increased worldwide and is among the top 10 causes of death and morbidity in the world [1]. In people with diabetes, there is an increased incidence of heart failure regardless of how coronary artery disease and hypertension are controlled [2]. Diabetic Cardiomyopathy (DCM) is described as the presence of myocardial dysfunction without any other conventional cardiovascular risk factors such as overt clinical coronary artery disease, valvular heart disease, hypertension, and dyslipidemia. It is a result of cardiomyocyte hypertrophy, and extracellular matrix arrangement deviations, and enhanced cardiac fibrosis. Studies have shown that mitochondrial dysfunction, increases in oxidative stress, and inflammation are all involved in the development and progression of diabetic cardiomyopathy [3].

Oxidative stress can lead to the onset and complications of diabetes [4,5]. Recent studies have shown that derivatives of hyperglycemia, reactive oxygen, and nitrogen species (ROS and RNS) can induce oxidative stress, which causes abnormal gene expression, altered signal transduction, and the activation of pathways leading to the death of myocardial cells. This cell loss then contributes to the development of diabetic cardiomyopathy [6,7]. Chronic inflammation is closely associated with the development of DCM, after all, T2DM is the leading cause of cardiovascular disease and has long been classified as an inflammatory disease [8,9]. Hypertrophic growth of the heart may occur as an adaptive response to hemodynamic stress or as a result of exercise or pregnancy, in which case it is deemed mild and reversible [10–12]. However, under chronic stressful conditions, there will be pathological hypertrophy with an excessive increase in ventricular dimensions, accompanied by myocardial dysfunction and fibrosis [13,14], which are characteristics of DCM.

There are many possible mechanisms for the development of DCM, including oxidative stress and inflammation, as well as mitogen-activated protein kinase-mediated signaling pathways that are common among these pathogenic responses. These mitogen-activated protein kinases (MAPK) play an important role in the development of heart failure, including diabetic cardiomyopathy. There has been much focus on p38 MAPK because it can promote or inhibit the translation of target genes [15,16]. p38 MAPK modulates genes that regulate cell hypertrophy, myocyte apoptosis, cardiac fibrosis, and cardiac cytokine-mediated inflammation [17–19]. A study showed that inhibition of p38 MAPK activation could prevent the development of DCM. Therefore, p38 MAPK may be a diagnostic indicator and therapeutic target for DCM [15,16].

NF-κB is a pleiotropic transcription factor involved in the regulation of a variety of heart diseases. Although NF-κB signaling boasts a vital function in the physiology of cardiology, its role in diabetic cardiomyopathy is still being pursued [20,21]. Pharmacological approaches have been taken in investigating its role in diabetic cardiac dysfunction but have proven to lack specificity. In addition, in diabetes, the exact cellular pathways in which NF-κB regulates heart function have not yet been determined. However, in a study to investigate the role of NF-κB signaling in diabetes-induced cardiac dysfunction using a genetic model of NF-κB inactivation, the results showed that the absence of NF-κB activation prevents diabetic cardiomyopathy development in animals [22]. Pathologically, cardiac hypertrophy, ischemia-reperfusion injury, myocardial infarction, and diabetic cardiomyopathy exhibit NF-κB activation [23,24].

Humanin (HN) is a peptide with cytoprotective properties in many cell types to resist stress. *In-vitro* and *in-vivo* studies have shown that HN significantly suppressed apoptosis when treating bone osteoporosis, neurodegenerative diseases such as Alzheimer’s disease, cardiovascular diseases, and diabetes mellitus. It has been suggested that the development of another mitochondrial-derived peptide, along with HN, might provide a potential treatment for different oxidative stress and apoptosis-related diseases [25,26]. S14G-humanin (HNG), is a potent humanin analogue. It is produced by substituting glycine for Ser14. This substitution can effectively increase the activity of Humanin.

HNG has been shown to be cytoprotective in male germ cells and leukocytes exposed to the toxic effects of chemotherapy. Its neuroprotective effects against oxygen-glucose deprivation/reoxygenation (OGD/R), characteristic of ischemic stroke, have also been investigated and proven [26]. Furthermore, HNG proved to possess cytoprotective effects on mouse epidermal stem cells upon ultraviolet (UV)-B treatment.

However, the pharmacological function of HNG in diabetic cardiomyopathy has not yet been reported. In the present study, we established diabetic mice models via stimulation with Streptozotocin (STZ), a naturally occurring alkylating antineoplastic agent that is particularly toxic to the insulin-producing β cells of the pancreas and is widely used in type 2 diabetes models [27,28]. We aimed to investigate the protective effects of S14G-humanin (HNG) against the resulting cardiac dysfunction.

## 2. Materials and methods

### 2.1 Animal model

Forty adult C57BL/6 mice (male, 2 months old) were the experimental animal choice for this study. Mice were purchased from Vital-Aiver Animal Ltd (Beijing, China). Procedures were done according to the Guide for the Care and Use of Laboratory Animals and Use Committee of Hainan Medical University. Procedures were approved by the Ethics Committee of the institute. Diabetes was induced in the mice; this was done by injecting intraperitoneal 50 mg/kg of STZ (AbMole, Houston, USA). The control group was injected intraperitoneal with Saline. After the period of induction of diabetes, blood samples were collected from the tail vein of the mice with a lance prick, and the blood glucose concentration was measured with a glucose monitor. Mice with hyperglycemia of >15 mmol/L after STZ were considered to be diabetic. Thereafter, the mice were divided into four groups (ten mice in each group): the control group (treated with saline IP), the HNG group (treated with 100 mg/kg HNG 0.5 ml saline IP), the DM group (treated with STZ in 0.1 m sodium citrate (PH 4.5) at a dose of 50 mg/kg) and the DM + HNG group (treated with 50 mg/kg) STZ and 100 mg/kg HNG (GenScript, New Jersey, USA) in 0.5 ml saline IP [29,30].

### 2.2 Assessment of heart function

Echocardiographic imaging was used to measure the heart function of the mice. 2D ECHO with Vevo 2100 (Visual Sonics Inc, Toronto, Ontario, Canada) was performed on unconscious mice. Isoflurane (Topscience, Shanghai, China) was used to anesthetize the mice. The heart rates of the mice were constantly monitored and kept between 400 and 500 beats per minute. The chests of the mice were shaved, and to maintain body temperature, they were placed on a warm pad. Prewarmed echo transmission gel was applied to the shaved areas. M-mode images of the left ventricle (LV) in both the long and short axis were recorded. Heart images were recorded to obtain left ventricle fractional shortening % (LVFS), left ventricle ejection fraction % (LVEF), and heart rate. The following data were recorded as the average of 3 consecutive cardiac cycles: the left ventricular end-diastolic volume (LVEDV), LV end-systolic volume (LVESV), LV end-diastolic dimension (LVEDD), and LV end-systolic dimension. LVFS was measured as follows: LVFS = (LVEDD−LVESD/LVEDD) ×100%. LVEF = (LVEDV – LVESV)/LVEDV×100%. The echo gel residues were removed, and mice were left to recover. Images were analyzed on a Vevo LAB image analysis workstation.

### 2.3 Preparation of myocardial tissue and blood samples

All mice were euthanized with an overdose of isoflurane at the end of the treatment period. Blood samples were drawn, and were mice dissected. The hearts were extracted, and the left ventricle was cut into 1 mm-thick sections. Tissues were kept at a low temperature of 4°C for further examination [31].

### 2.4. Wheat Gem Agglutinin (WGA) staining and quantification

WGA staining was used to analyze cardiomyocyte size. The specimen was fixed (10% formalin), embedded in paraffin, and cut into sections. After being de-waxed and rehydrated, 5 μm myocardial slides were boiled in sodium citrate buffer (10 mM) for 10 minutes and blocked with 1% BSA for 1 hour. Slides were then stained with 5 μM AlexaFluor-594 conjugated WGA (Invitrogen, USA). Results were quantified using Image J (NIH, USA).

### 2.5. Measurement of indicators relevant to myocardial oxidative stress and antioxidant capacity

Commercial kits were used to determine TBARS level (Cambridge, USA), NOX activity (Solarbio, Beijing, China), The Catalase Level (Abcam, Cambridge, UK), SOD activity (Abcam, Cambridge, UK), and GPx levels (Fusheng, Shanghai, China) in accordance with the manufacturer’s instructions.

### 2.6. Detection of myocardial injury indicators

Blood samples were collected and centrifuged for 15 minutes at a speed of 2200–2500 RPM. Plasma was pipetted into separate tubes, CK-MB, AST, and LDH levels were analyzed with CK-MB ELISA kit (Senbeijia, Nanjing, China), AST kit (Sigma-Aldrich, St Louis, USA), and LDH kit (Abcam, Cambridge, UK). The manufacturer’s instructions were followed in all test kits used.

### 2.7. ELISA assay for TNF-α and IL-6 in Cardiac tissue and Plasma

Blood collection was done after the mice abstained from food for 12 hours. The samples were put in a centrifuge (3000RPM) for 10 minutes in order to obtain Plasma. Thereafter incubation followed for 15 minutes at room temperature. The samples were kept in the freezer. Levels of TNF-α and IL-6 proteins in plasma and tissues were measured with ELISA kits (R&D Systems), and these were done according to instructions given by the manufacturer.

### 2.8. Western blot analysis

Tissues were washed in normal saline and homogenized in cell lysis buffer in ice. The lysates were pipetted into microcentrifuge tubes and centrifuged (15000RPM for 30 minutes). An inhibitor cocktail was added, and the samples incubated. Subsequently, the tubes were centrifuged, and 10 μg of the protein mix was separated on 10% SDS PAGE and transferred to PVDF membranes, then blocked using 5% skim milk in TBST for 1 hour. Incubation overnight was done after adding primary antibodies, followed by washing, adding secondary antibodies, and quantifying and calculating the results [32].

### 2.9. Statistical analysis

Results were shown as mean± standard deviation (S.D.). The comparison was analyzed using analysis of variance followed by Bonferroni’s test. A P value of less than 0.05 was considered statistically significant.

## 3. Results

Using the *in vivo* mice models, we investigated the effects of HNG on STZ-induced cardiac dysfunction. We examined the levels of CK-MB, AST, and LDH, as well as the cardiac and plasma levels of TNF-α and IL-6. To understand the underlying mechanism, we further tested the effect of HNG on STZ-induced activation of the p38/NF-κB signaling pathway.

### 3.1. Protective effects of S14G-humanin in basic parameters of diabetic mice

**Figure 1.**
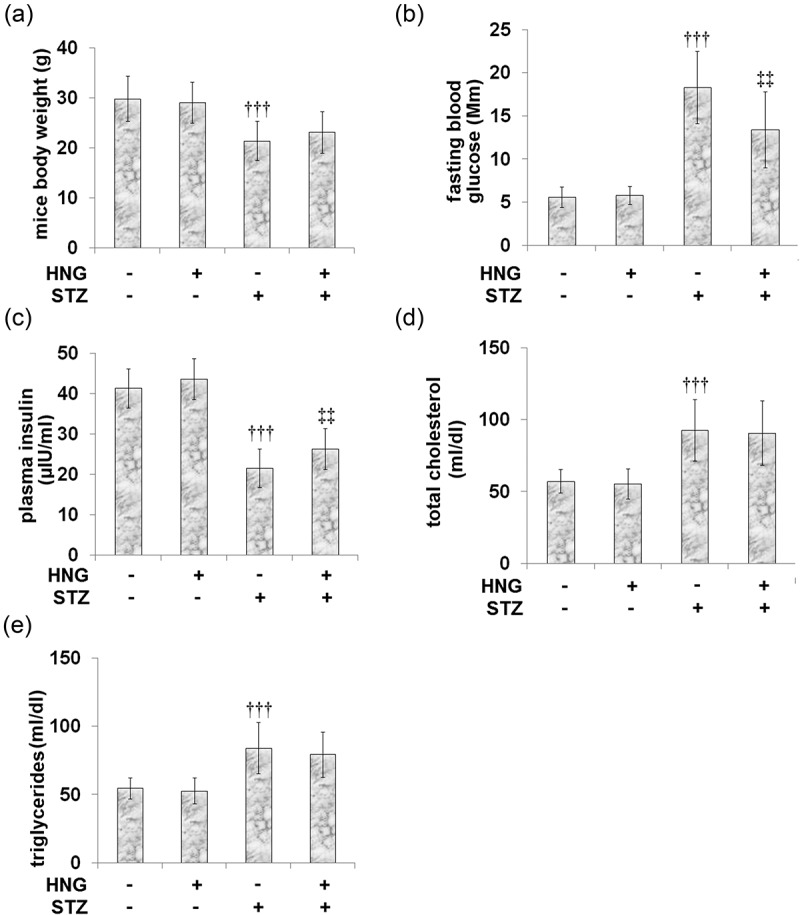
Protective effects of S14G-humanin in basic parameters of diabetic mice. (a) mice body weight; (b) Fasting blood glucose; (c) plasma insulin; (d) Total cholesterol; (e) Triglycerides (†††, P < 0.005 vs. vehicle group; ‡‡, P < 0.01 vs. STZ group)

Figure 1 shows that when compared to the control, the STZ-diabetic mice showed lower plasma insulin levels (Figure 1c) and body weight (Figure 1a). On the other hand, fasting glucose concentration (Figure 1b), total cholesterol (Figure 1d), and triglycerides (Figure 1e) were increased. Following the introduction of HNG to both the control and DM groups, no significant effect was observed on the basic parameters of the mice in either group, indicating that its effects on diabetic cardiomyopathy might be independent of glycemic control.

### 3.2. Protective effects of S14G-humanin on myocardial hypertrophy in diabetic mice

**Figure 2.**
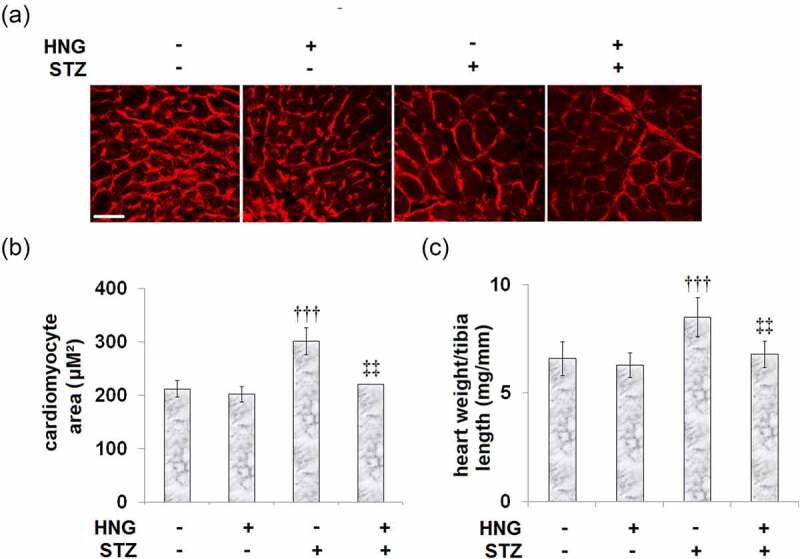
Protective effects of S14G-humanin on myocardial hypertrophy in diabetic mice. (a). Representative images of WGA staining in the cardiomyocyte cross-sectional area; Scale bar, 100 μM; (b). Quantification analysis of Cardiomyocyte area; (c). Heart weight/tibia length (†††, P < 0.005 vs. vehicle group; ‡‡, P < 0.01 vs. STZ group)

The study is premised on the realized involvement of myocardial hypertrophy in the occurrence and development of diabetic cardiomyopathy [3], so we investigated the effect of HNG on the STZ-induced myocardial hypertrophy. Figure 2a shows the representative images of WGA staining in the cardiomyocyte cross-sectional area. Compared to the control, the STZ-diabetic mice had an increased cardiomyocyte area and heart weight/tibia length, as shown by Figure 2b and C, respectively. Treatment of these diabetic mice with HNG reduced both the cardiomyocyte area and heart weight/tibia length, indicating that HNG treatment attenuated the STZ-induced myocardial hypertrophy in the mice. On the other hand, in the control mice, treatment with HNG had no significant effect on neither the cardiomyocyte area nor the heart weight/tibia length.

### 3.3 Protective effects of S14G-humanin on heart function

**Figure 3.**
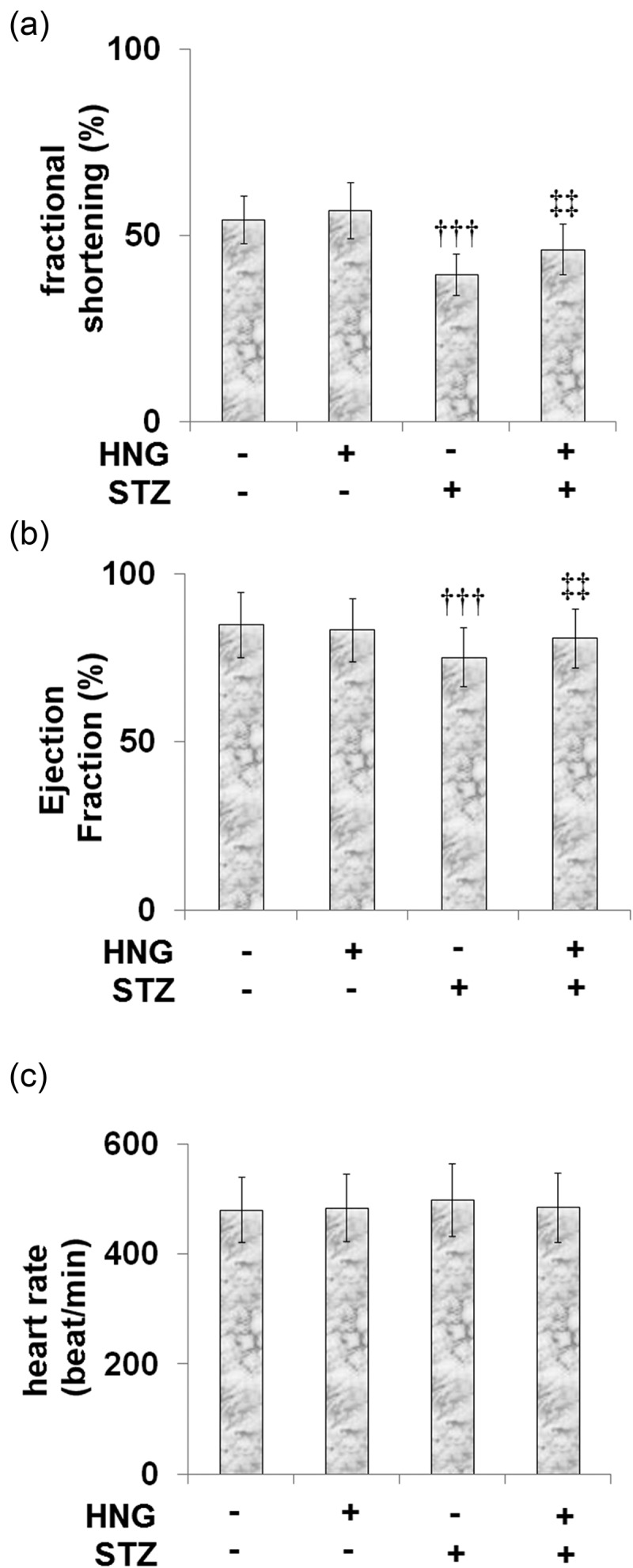
Protective effects of S14G-humanin on heart function. The mice heart function was measured by Echo. (a) Fractional shortening;(b) Ejection Fraction; (c) heart rate (†††, P < 0.005 vs. vehicle group; ‡‡, P < 0.01 vs. STZ group)

We broadened our experiments to investigate the therapeutic potential of HNG for STZ-induced heart malfunction. STZ prompted decreased fractional shortening in the mice, from 54.2 ± 6.3% in the control to 39.5 ± 5.6% (Figure 3a). Treatment with HNG alleviated this decrease to 46.2 ± 6.8%. Likewise, HNG treatment resulted in an ejection fraction of 80.8 ± 8.9%, ameliorating the STZ-induced decrease in ejection fraction from 84.9 ± 9.7% of the control to 75.2 ± 8.7% (Figure 3b). Also, the heart rates of the control, HNG, DM, and DM + HNG mice groups were 480.6 ± 59.7, 483.6 ± 61.1, 498.5 ± 65.8 and 485 ± 62.8 bpm (Figure 3c), showing that heart function was significantly improved by the introduction of HNG. HNG did not have a noteworthy consequence on the heart function in the control mice.

### 3.4 Effects of S14G-humanin on the myocardial injury indicators in the control and diabetic mice

**Figure 4.**
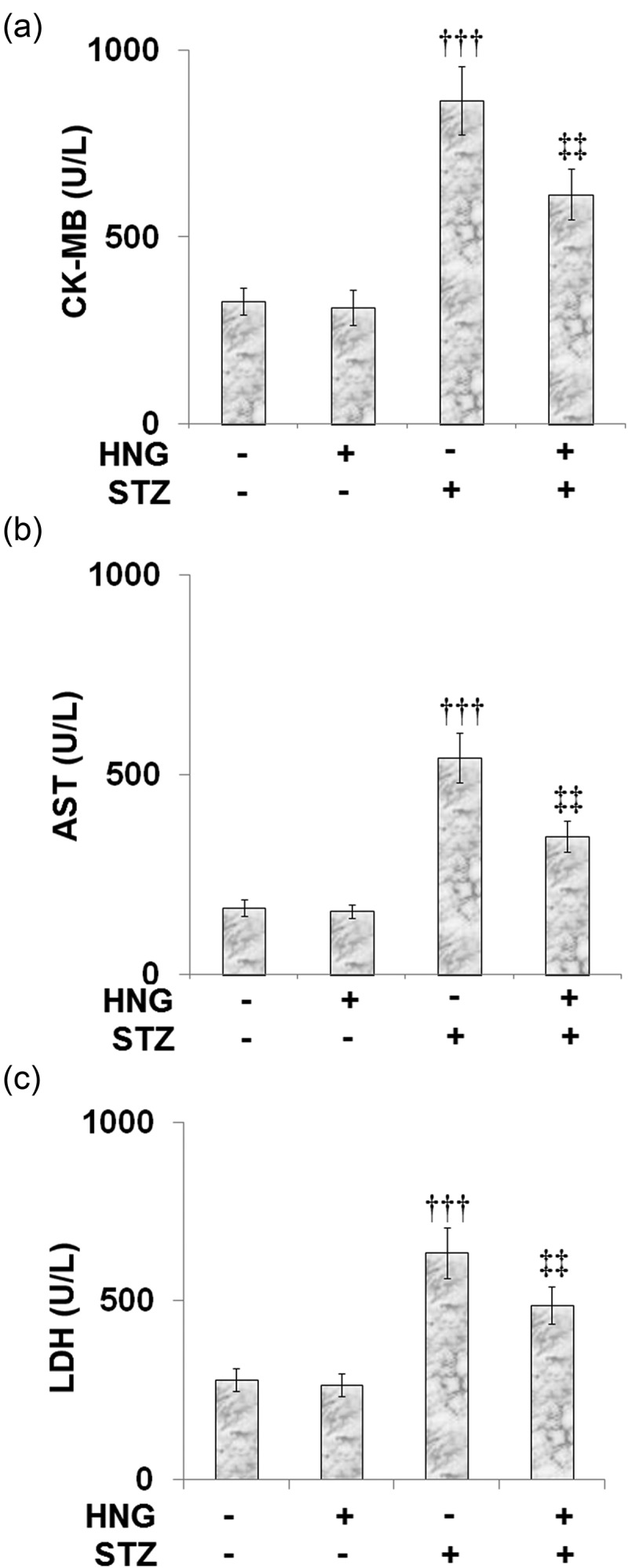
Effects of S14G-humanin on the myocardiac injury indicators in the control and diabetic rats. (a) The CK-MB Level; (b) The AST Level; (c) The LDH Level (†††, P < 0.005 vs. vehicle group; ‡‡, P < 0.01 vs. STZ group)

AST, LDH, and creatine kinase-MB (CK-MB) are some of the indicators for the diagnosis of acute myocardial injury [33,34] because they are released from cardiac muscle cells following injury. In our experiment, compared to the control, the STZ-diabetic mice exhibited significantly increased CK-MB (Figure 4a), AST (Figure 4b), and LDH (Figure 4c) levels, indicating myocardial injury. Treatment of the diabetic mice with HNG proved effective as it reduced the levels of all the myocardial injury indicators, highlighting the effect of HNG on the STZ-induced myocardial injury. Treating the control mice with HNG had no significant effect on the levels of CK-MB, AST, and LDH, suggesting that HNG treatment alone had no side effects.

### 3.5 S14G-humanin ameliorated the inflammation-related genes induced by STZ in diabetic mice

**Figure 5.**
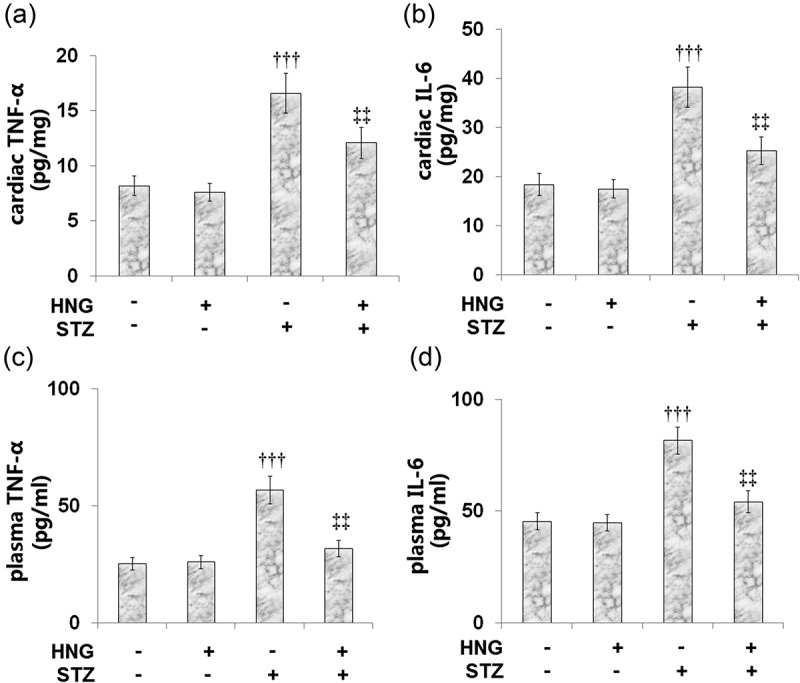
S14G-humanin ameliorated the inflammation-related genes induced by STZ in diabetic mice. (a). Cardiac TNF-α Level; (b). Cardiac IL-6 Level; (c). Plasma TNF-α Level; (d). Plasma IL-6 Level (†††, P < 0.005 vs. vehicle group; ‡‡, P < 0.01 vs. STZ group)

TNF-α and IL-6 are some of the main proinflammatory cytokines involved in the development and progression of diabetic complications [35]. Therefore, we examined the effect of HNG on the levels of these proinflammatory cytokines. Figure 5a and b show the cardiac levels of TNF-α (8.2 ± 0.9, 7.6 ± 0.81, 16.6 ± 1.8, and 12.1 ± 1.4 pg/mg) and IL-6 (18.4 ± 2.2, 17.5 ± 1.9, 38.2 ± 4.1, and 25.3 ± 2.8 pg/mg) in the Control, HNG, DM, and DM + HNG mice groups, respectively. These results show that STZ- induced increases in the cardiac levels of the aforementioned proinflammatory cytokines. However, the treatment with HNG proved therapeutic as it decreased these levels. Similarly, STZ treatment increased the plasma level of TNF-α from 25.3 ± 2.8 to 56.7 ± 5.9 pg/ml, which was later reduced to 31.7 ± 3.5 pg/ml following the introduction of HNG. The plasma IL-6 level was also initially increased from 45.5 ± 3.8 to 81.6 ± 6.1 pg/ml by STZ alone, then later reduced to 54.2 ± 4.9 pg/ml by HNG. The effect of HNG treatment on neither the cardiac nor plasma levels of TNF-α and IL-6 in the control mice proved significant.

### 3.6 S14G-humanin ameliorated oxidative stress in cardiac diabetic mice

**Figure 6.**
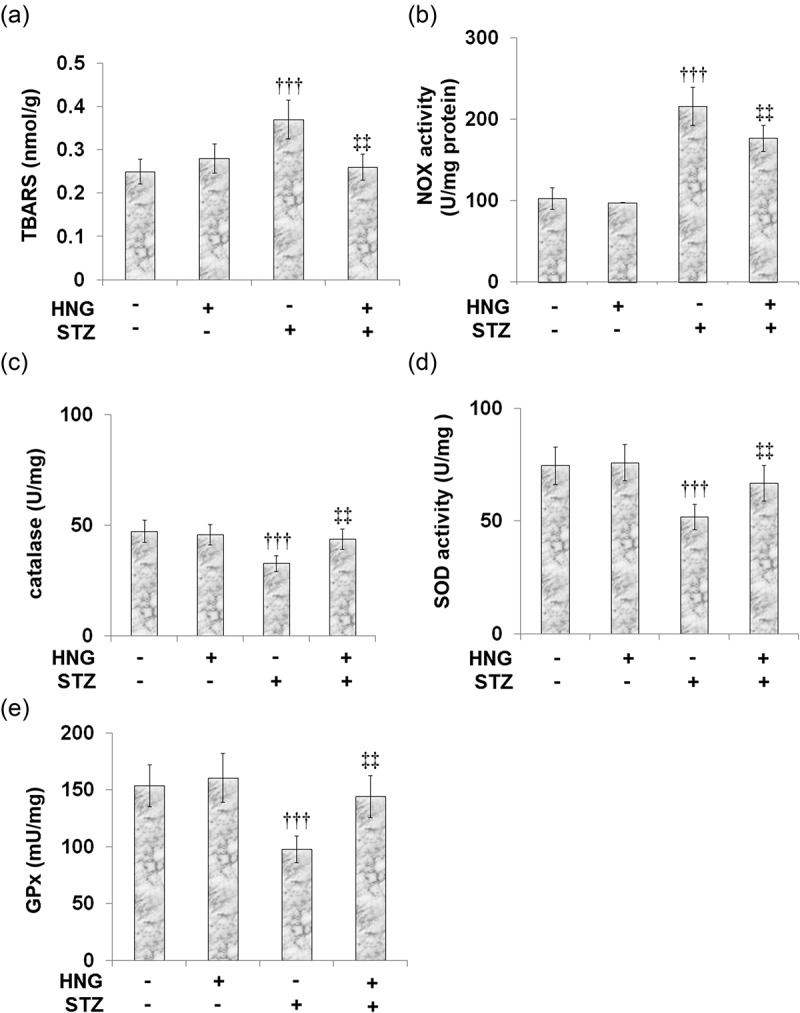
S14G-humanin ameliorated oxidative stress in cardiac diabetic mice. (a) TBARS level; (b) NOX activity; (c) The Catalase Level; (d) SOD activity; (e) GPx levels (†††, P < 0.005 vs. vehicle group; ‡‡, P < 0.01 vs. STZ group)

Oxidative stress is a key contributor to the mechanism underlying the pathophysiology of diabetes-induced cardiac dysfunction. We examined the therapeutic potential of HNG on the oxidative stress induced by STZ using the levels and activities of the protective antioxidant enzymes superoxide dismutase (SOD), catalase and glutathione peroxidase (GPx), as well as the oxidative stress markers thiobarbituric acid reactive substances (TBARS), and NADPH-oxidase (NOX), which is responsible for the production of ROS. Figure 6a shows the TBARS level being increased from 0.25 ± 0.029 to 0.37 ± 0.045 nmol/g by STZ, then later being reduced to 0.26 ± 0.03 nmol/g after the introduction of HNG. Likewise, STZ increased the NOX activity from 102.6 ± 13.5 to 215.7 ± 23.6 U/mg protein, which was then reduced to 176.6 ± 16.1 U/mg protein by the treatment with HNG (Figure 6b). On the other hand, the catalase level was decreased by STZ, from47.3 ± 4.9 to 32.7 ± 3.5 U/mg, then increased to 43.8 ± 4.6 U/mg by the treatment with HNG (Figure 6c). Similarly, the SOD activity was reduced from 74.6 ± 8.3 to 51.8 ± 5.6 U/mg by STZ treatment and then increased to 66.7 ± 7.9 U/mg by the introduction of HNG (Figure 6d). The GPx levels were also reduced by STZ, from 153.8 ± 18.4 to 97.8 ± 11.6mU/mg, then later increased to 144.2 ± 18.3 mU/mg by the treatment with HNG (Figure 6e). These results show that HNG indeed ameliorated STZ-induced oxidative stress, although it had little effect on the oxidative stress in the control mice.

### 3.7 Protective effects of S14G-humanin on p38 MAPK and NF-κB signaling pathway in cardiac diabetic mice

**Figure 7.**
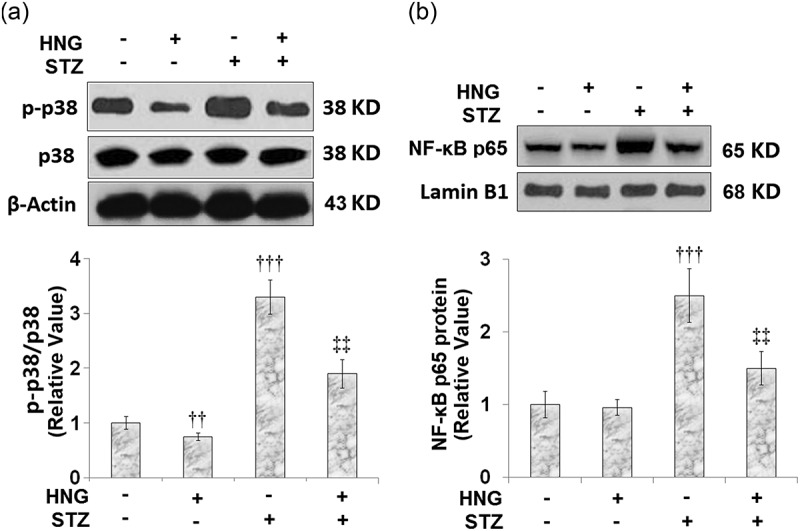
Protective effects of S14G-humanin on p38 MAPK and NF-κB signaling pathway in cardiac diabetic mice. (a) Protein expression of Phosphorylated p38/p38; (b) Protein expression of nuclear NF-κB p65 (†††, P < 0.005 vs. vehicle group; ‡‡, P < 0.01 vs. STZ group, n = 7–8)

We extended our experiments to investigate the effects of HNG on the proinflammatory pathways, p38 MAPK and NF-κB. Compared to the control, the STZ-diabetic mice had increased protein expressions of p-p38/p38 (Figure 7a) and nuclear NF-κB p65 (Figure 7b). The introduction of HNG had inhibitory effects on the expressions of phosphorylated p38/p38 and nuclear NF-κB p65, evidenced by their reduced protein expressions, highlighting the protective effects of HNG on p38 and the NF-κB signaling pathway in cardiac diabetic mice. HNG did not have a significant effect on the Phosphorylated p38/p38 and NF-κB signaling in the control mice.

## 4. Discussion

Among diabetic patients, diabetic cardiomyopathy is one of the main causes of morbidity and mortality. It is primarily characterized by myocardial fibrosis and associated diastolic dysfunction, along with the development of cardiomyocyte apoptosis and cardiac hypertrophy. Although the pathophysiological mechanism of cardiac dysfunction caused by diabetes is quite complex and multi-factorial, it is understood that oxidative stress is a key factor [3]. The experimental evidence that ROS is a predecessor of diabetic cardiac complications stemmed from reports evaluating the rate of lipid peroxidation. In mice treated with streptozotocin (STZ), elevated cardiac levels of the widely used oxidative stress markers, thiobarbituric acid reactive substances (TBARS), and lipid peroxides were observed [36,37]. NADPH-oxidases (NOXs) are prominent ROS-producing enzymes in cardiomyocytes, which mediate both adaptive and maladaptive changes, and have been suggested as a therapeutic target for various diabetic complications [38]. When free radicals like the superoxide anion and hydrogen peroxide exceed the body’s ability to regulate them, oxidative stress follows. Cells are protected by enzymes such as superoxide dismutase (SOD) and catalase from such radical attacks [39,40]. Including glutathione peroxidase (GPx), they are first-line defense antioxidants and are very important and indispensable in the entire defense strategy of antioxidants, as highlighted by the many publications concerning antioxidants and their significance in preventing oxidative stress and the resulting cellular damage [41]. In our current study, following stimulation with STZ, we showed that S14G-humanin ameliorated the resulting oxidative stress in cardiac diabetic mice, evidenced by the decreased TBARS level and NOX activity and increased SOD activity, catalase, and GPx levels.

The underlying mechanisms of diabetic heart dysfunction are complex and involve multiple molecular phenotypes, including activation of p38 MAPK [42] and proinflammatory states. The pathogenesis of diabetes and its complications are related to chronic low-grade inflammation and activation of the innate immune system. TNF-α and IL-6 are some of the main proinflammatory cytokines involved in the development and progression of diabetic complications [35]. One study evaluated the proinflammatory cytokine levels (TNF-α, IL-6) in left ventricular diastolic dysfunction (LVDD), the earliest manifestation of diabetes-induced left ventricular dysfunction, and the result was increased plasma levels of IL-6 and TNF-α [43]. Consistent with this, our experiments showed that the levels of cardiac and plasma IL-6 and TNF-α increased after STZ stimulation in diabetic mice. These increased levels were later remedied by the introduction of HNG. p38 MAPK is activated in response to inflammatory cytokines. The p38 MAPK pathway regulates the synthesis of proinflammatory cytokines, making its different components a potential therapeutic target for inflammatory and autoimmune diseases [44]. NF-κB has long been regarded as a proinflammatory signaling pathway, based on its activation by proinflammatory cytokines and its role in the expression of other proinflammatory genes [45]. Currently, it is well established that NF-κB is a central inflammatory mediator that responds to a large variety of immune receptors. Its activation is involved in various inflammatory diseases; therefore, targeting the NF-κB signaling pathway could be a potential treatment approach to inflammatory diseases [40]. In this study, we sought to highlight the protective effects of S14G-humanin on p38 MAPK and the NF-κB signaling pathway in cardiac diabetic mice. Our results show that HNG reduced the protein expressions of NF-κB p65 and phosphorylated p38. When coronary artery flow decreases, ischemia ensues and leads to myocardial necrosis and deterioration of ventricular function [46]. Following necrosis, enzymes normally found in cardiac muscle cells are released. Therefore, enzymes such as ALT, AST, LDH, creatine kinase-MB (CK-MB), and troponins have been used as indicators for the diagnosis of acute myocardial injury [33,34]. In our experiments, we established myocardial injury in mice using STZ, and it was marked by increased levels of CK-MB, AST, and LDH. When we introduced HNG, these levels were markedly reduced, highlighting the protective effects of HNG on myocardial injury in diabetic mice. Following the establishment of STZ-induced cardiac dysfunction in our experiments, the basic heart function of the mice was impaired, as shown by the decreased fractional shortening and ejection fraction and increased heart rate. However, the treatment with HNG reversed these effects of STZ, indicating its protective role on the heart function of diabetic mice. Also, because myocardial hypertrophy is involved in the development and progression of diabetic cardiomyopathy [3], we tested for the effect of HNG on the myocardial hypertrophy in the diabetic mice and found that the cardiomyocyte area and weight of the heart were decreased.

## 5. Conclusion

In conclusion, based on the above findings, we conclude that HNG possesses protective effects against STZ-induced cardiac dysfunction through inhibiting the p38 MAPK/NF-κB signaling pathway. In the future, we will further provide more evidence for the application of HNG in the treatment of cardiac dysfunction.

## Data Availability

The data that support the findings of this study are available from the corresponding author upon reasonable request.
